# Juvenile Myasthenia Gravis: A Paediatric Perspective

**DOI:** 10.4061/2011/404101

**Published:** 2011-11-01

**Authors:** Maria F. Finnis, Sandeep Jayawant

**Affiliations:** Department of Paediatric Neurology, Children's Hospital, John Radcliffe Hospital, Oxford OX3 9DU, UK

## Abstract

Myasthenia gravis (MG) is an autoimmune disease in which antibodies are directed against the postsynaptic membrane of the neuromuscular junction, resulting in muscle weakness and fatigability. Juvenile myasthenia gravis (JMG) is a rare condition of childhood and has many clinical features that are distinct from adult MG. Prepubertal children in particular have a higher prevalence of isolated ocular symptoms, lower frequency of acetylcholine receptor antibodies, and a higher probability of achieving remission. Diagnosis in young children can be complicated by the need to differentiate from congenital myasthenic syndromes, which do not have an autoimmune basis. Treatment commonly includes anticholinesterases, corticosteroids with or without steroid-sparing agents, and newer immune modulating agents. Plasma exchange and intravenous immunoglobulin (IVIG) are effective in preparation for surgery and in treatment of myasthenic crisis. Thymectomy increases remission rates. Diagnosis and management of children with JMG should take account of their developmental needs, natural history of the condition, and side-effect profiles of treatment options.

## 1. Introduction

Myasthenia gravis (MG) is an autoimmune disease in which antibodies are directed at the postsynaptic membrane of the neuromuscular junction, leading to varying degrees of muscle weakness and fatigability. Where MG presents before 19 years of age, it is termed juvenile myasthenia gravis (JMG). Although JMG shares many features with the more common adult MG, there are many important differences. 

In this paper we discuss the pathogenesis, epidemiology, presentation, treatment, and outcome of JMG and highlight some of the clinical features and challenges particular to paediatric patients.

## 2. Pathogenesis

In the majority of cases MG is caused by antibodies to the nicotinic acetylcholine receptor (AChR). Antibodies to the AChR are found in over 80% adults with generalised disease but only in 55% of adults with weakness confined to the oculomotor muscles. Patients with AChR antibodies are often referred to as seropositive. AChR antibodies are probably less frequent in prepubertal patients than in adolescent and adult patients [1, 2] (see Table 1). Antibodies to muscle-specific kinase (MuSK) and to Leucine rich protein 4 (LRP4) have been reported in some seronegative patients. 

Childhood myasthenias encompass JMG, which is the subject of this paper; congenital myasthenic syndromes, a heterogeneous group of genetically inherited disorders of the neuromuscular junction [3]; transient neonatal myasthenia, which results from placental transfer of maternal AChR (or very occasionally MuSK antibodies) to infants of mothers with autoimmune MG [4].

## 3. Epidemiology and Clinical Features

JMG is a rare disorder of childhood, but its incidence and prevalence vary geographically. Precise data on incidence and prevalence are not known. Paediatric presentation of MG is more common in Oriental than in Caucasian populations [5]. Up to 50% of all cases of MG in Chinese populations present in childhood, mostly with ocular features, with a peak age at presentation of 5–10 years [6]. Caucasian patients, in contrast, are more likely to present in adulthood [7, 8], with prepubertal onset in less than 10% cases [2, 9].

The most frequent clinical presentation of JMG is with ptosis, which is often associated with other ocular symptoms namely unilateral or asymmetric ophthalmoplegia, strabismus, and lid twitch, which may only be elicited after sustained upgaze [10]. These symptoms cause particular problems in children as, if severe, they may cause persistent amblyopia [11]. Most children also develop generalised muscle weakness, which presents as painless fatigability of the bulbar and limb musculature, with resultant dysphonia, dysphagia, and proximal limb weakness. Weakness is often fluctuating and usually becomes more pronounced through the day and improves with rest. Children are at risk of choking or aspiration and are at increased risk of chest infection. Occasionally, impairment of the respiratory muscles necessitates ventilatory support. This is known as “myasthenic crisis”.

Prepubertal children presenting with JMG have some interesting and distinct clinical features compared with those who present around or after puberty [1, 2]. Prepubertal JMG is more likely to manifest as ocular myasthenia [12]. There is an equal male: female ratio [13], in contrast to the female predominance that is seen in peri-/postpubertal children, and a better prognosis, with a higher rate of spontaneous remission in prepubertal presenters [1, 12]. Peri- or postpubertal patients presenting with JMG share more similarities with adult-onset MG (see Table 1).

Ocular myasthenia gravis (OMG) is, by definition, MG restricted to the oculomotor muscles for 2 years without becoming generalised [14]. In adult populations up to 80% patients with OMG at presentation will progress to generalised disease [8, 9, 15]. Case series in children (using a variety of treatment protocols and follow-up intervals) have reported lower rates of generalisation than adults [16]. Progression may be even less frequent in prepubertal children [17, 18].

### 3.1. Transient Neonatal Myasthenia

This results from transfer of maternal AChR antibodies across the placenta leading to defects of neuromuscular transmission in the neonate [4]. Not all mothers have detectable AChR antibodies and a few are asymptomatic at the time. Usually the affected baby is normal at birth, subsequently developing signs such as hypotonia, weak cry, poor suck, reduced movements, ptosis and facial weakness, and occasional respiratory insufficiency requiring mechanical ventilation. Short-term treatment with anticholinesterases is usually sufficient.

## 4. Diagnosis of JMG

JMG is primarily a clinical diagnosis with classical patterns of fluctuating weakness and fatigability as described above. A number of diagnostic tools are available to aid with diagnosis. In very young children it is particularly important to distinguish between autoimmune myasthenia and congenital myasthenic syndromes (CMS) as the treatment options, prognosis, and genetic implications are very different (see Table 2). 

CMS usually present in the first years of childhood with variable disability. There is often a positive family history, and diagnosis is aided primarily by electrophysiology and DNA analysis and occasionally by muscle biopsy [21]. With the exception of the autosomal dominantly inherited slow-channel syndrome, the CMS are inherited by autosomal recessive mutations, which result in loss of function at the neuromuscular junction [10]. 

### 4.1. Serology

Detection of antibodies to the AChR supports the diagnosis of JMG. In young children where AChR antibodies are negative this can lead to difficulty in differentiating from CMS. Some of these children who are negative for AChR antibodies will have “low affinity” antibodies to the AChR which were not detectable using the standard assays [22]. Some children will, in fact, turn out to have CMS.

A variable percentage (0–49%) of MG patients without AChR antibodies are found to have antibodies against another neuromuscular junction protein, the muscle-specific kinase (MuSK) [23]. MuSK positive MG is rare in children, and these children represent a distinct subgroup of JMG, with a marked female predominance. MuSK antibodies appear to be associated with more severe disease with prominent facial and bulbar weakness and frequent respiratory crises [24].

Patients without antibodies to AChR or MuSK are described as having seronegative myasthenia gravis (SNMG). SNMG patients are phenotypically more similar to AChR seropositive patients than MuSK positive patients, both in clinical presentation and in response to treatment. “Low affinity” antibodies to clustered AChRs can be found in 60% of previously defined SNMG patients. These antibodies are found in all age groups [22]*.*

Seroconversion has been described in a small number of cases of children who have developed MuSK antibodies after thymectomy for AChR seropositive MG [25]. This has not been described in adults.

Other potential antigens at the neuromuscular junction have been identified in adults with later-onset MG, but the relevance to the childhood population has not been established [26].

### 4.2. Pharmacological Investigation

The Tensilon test involves intravenous infusion of edrophonium, a fast-acting, short-duration cholinesterase inhibitor. This prevents the breakdown of acetylcholine, thereby increasing the concentration of the neurotransmitter at the neuromuscular junction. The patient is observed, and ideally a video recorded, looking for a transient improvement in previously documented weakness, for example, ptosis, dysphonia. This test is not without risk and should only be performed by staff experienced in paediatric resuscitation, due to the cholinergic effects of edrophonium, which can result in bradycardia, nausea, and excess salivation. 

### 4.3. Electrophysiology

Electrophysiological testing can be invaluable in investigation of suspected JMG. Repetitive nerve stimulation in JMG will show a decrement in the compound motor action potential of >10% by the 4th or 5th stimulation.

Single fibre EMG (SFEMG) is especially useful in diagnosis of seronegative MG and congenital myasthenic syndromes. It can be technically more difficult in children due to discomfort of the procedure and the level of cooperation required. It can be done under local or even general anaesthetic. Sensitivity for a neurotransmission disorder is 97% [27]. A normal result therefore makes a diagnosis of myasthenia very unlikely [28]. 

### 4.4. Imaging

Although thymoma in children is rare, the thymus must be imaged (usually by CT) once JMG has been diagnosed. AChR seropositive MG is frequently associated with changes in the thymus, with histological changes and in vitro effects suggesting that the thymus plays a pathogenic role [47]. Thymus hyperplasia is the commonest abnormality of the thymus in JMG [20]. Thymoma is particularly rare in prepubertal children [12]*.*

Thymus changes are not a common feature of MuSK positive disease, and thymoma has not been reported in MuSK-positive children.

Thymus abnormalities in SNMG patients have been found to be histologically very similar to the thymus hyperplasia seen in AChR seropositive MG [47].

## 5. Management

Management of children with JMG should be delivered by a multidisciplinary team comprising a paediatrician with support from a paediatric neurologist, physiotherapist, occupational therapist, psychologist, speech therapist and dietician. Other members of the team may also need to be involved, depending on associated comorbidities such as bulbar weakness leading to difficulty with oral feeding, or respiratory insufficiency requiring noninvasive ventilatory support, which should be managed by a respiratory paediatrician.

Treatment of JMG has largely been extrapolated from adult studies and experience with adult patients. There are few studies looking specifically at interventions in children, particularly prepubertal children (see Table 3). Some case series include paediatric patients but they are often not subdivided into prepubertal and postpubertal age groups for analysis. Given the evidence that prepubertal JMG may behave quite differently in terms of disease severity and progression, this may impact on necessity for treatment and treatment response. Side-effect profiles and considerations are not always directly comparable between adult and paediatric populations.

### 5.1. Acetylcholinesterase Inhibitors

Acetylcholinesterase inhibitors are first-line treatment in JMG and provide symptomatic relief. In mild cases and in some cases of ocular MG, acetylcholinesterase therapy may be sufficient. Pyridostigmine is a long-acting cholinesterase inhibitor that is commonly used. Dosing is usually 4–6 times per day and is tailored to effects. Cautious use in MuSK-positive children is advised due to risk of acetylcholine hypersensitivity [48].

### 5.2. Thymectomy

Because of the presumed role of the thymus in the pathogenesis of MG, thymectomy is a recognised aspect of management. Thymectomy may remove thymic germinal centres and disrupt antibody diversification [47]. A systematic review of the literature concluded that thymectomy increases the probability of remission or improvement of symptoms in AChR seropositive, nonthymomatous, autoimmune MG [49]. This paper included only one paediatric study [31]. More recent reviews of children including prepubertal patients, also suggested increased remission rates after thymectomy [30, 32]. Caution needs to be taken in early childhood due to subsequent immunosuppression and the high rates of spontaneous remission in prepubertal presenters.

Current evidence suggests that thymectomy should not be recommended in MuSK-positive disease as it is unclear whether it confers any benefit [29, 50, 51]. 

Thymectomy in pure OMG remains controversial. Whereas OMG is not life threatening, patients may be dependent on long-term immunosuppressant medications, including corticosteroids, with the resultant side effects which can be substantial in children. Persistent amblyopia can result in children as the visual system is maturing. As discussed above, we know that a proportion of children will progress to generalised disease. Thymectomy is not proven to reduce risk of progression of OMG to generalised JMG [52] and is not routinely indicated in pure OMG in children but has been performed in refractory cases.

A variety of surgical methods for thymectomy have been described: full or partial sternotomy, thorascopic, or transcervical approaches. Evidence suggests that symptom resolution is equivalent regardless of surgical approach [53, 54]. After thymectomy there is increased risk of antimuscarinic side effects of cholinesterase inhibitors, and they should therefore be used under close supervision in the postoperative period.

### 5.3. Immunosuppressive Therapies

Frequently some form of immunosuppression or immunomodulation is required to improve symptoms of JMG. Corticosteroids are often effective and are the mainstay of therapy but can worsen symptoms in the first few weeks of use, particularly if started at high doses [33]. Because of the numerous adverse effects associated with long-term high-dose steroids, steroids are often used in combination with a steroid-sparing immunosuppressant, for example, azathioprine. Children are at particular risk of steroid side effects, including growth failure, susceptibility to severe infection, and delay in receiving live vaccinations.

Azathioprine is a purine analogue that suppresses B and T cell proliferation. It has been found to be effective when used alone [34], but is most commonly used in combination with prednisolone as a steroid-sparing agent. Beneficial effects may take months to be seen [35] but eventually result in weaning of steroid doses [36].

Some studies have suggested that azathioprine or corticosteroids may reduce the likelihood of progression of OMG to the generalised form of disease [55, 56]. Although these studies included some children in their case series, these were not specifically paediatric studies, and given the lower rates of progression in prepubertal children anyway, these findings are of uncertain relevance in paediatric practice.

Patients unresponsive or intolerant to azathioprine should be considered for other immunosuppressive agents, which could include cyclosporin A (which has a faster time to symptomatic benefit than azathioprine [37]) or cyclophosphamide [39]. A Cochrane review suggests that cyclosporin either as monotherapy or with corticosteroids, or cyclophosphamide in conjunction with corticosteroids, improves symptoms of MG within 1 year [38]. 

Mycophenolate mofetil (MMF) blocks purine synthesis by selectively inhibiting proliferation of activated T and B lymphocytes [57]. A recent retrospective study of AChR seropositive patients, which included children (age range 11–87 y), concluded some benefit of MMF when used either as monotherapy or in conjunction with prednisolone. Maximum effects may not be seen until after one year of treatment [43].

Tacrolimus inhibits interleukin 2. Efficacy studies have been carried out in adults and postpubertal children and have shown early and sustained improvement of symptoms with tacrolimus, allowing dose reduction of prednisolone and in many cases its complete withdrawal. These steroid sparing effects were seen within 6 months [40, 41]. A case has also been reported of tacrolimus being successfully used as adjunctive therapy in refractory pure ocular myasthenia in a 3-year-old girl [42]. 

Rituximab is a chimeric IgG monoclonal antibody that depletes B cells and has been used in refractory JMG [44]*.*

### 5.4. Plasma Exchange/Intravenous Immunoglobulin (IVIG)

Improvement in symptoms after plasma exchange or administration of IVIG is usually temporary, 4–10 weeks. Their use is therefore largely reserved to optimise condition for surgery before thymectomy and in management of myasthenic crisis [45, 46, 58]. A single randomised controlled trial showed no evidence for superior benefit of plasma exchange over IVIG in treatment of myasthenic crisis [59].

Efficacy studies are not available for prepubertal children. 

Allogeneic hematopoietic cell transplantation has been reported as successful in treating a 17-year-old male with refractory JMG that had been diagnosed aged 11 months [60].

## 6. Outcome

Outcomes in JMG have improved significantly over the last decade, with better recognition, diagnosis, and more effective therapies, and long-term prognosis is good [61]. Children with JMG exhibit higher rates of remission than adults. This includes spontaneous remission and remission following a period of drug therapy. Prepubertal children have the highest rates of spontaneous remission. Remission rates also appear to be influenced by ethnic origin [1].

## 7. Summary

JMG is a rare, autoimmune condition of childhood that shares many characteristics of clinical presentation and management strategies with the adult form of the disease. However, as described in this paper, there are many important aspects that are specific to the paediatric population, in particular the distinct clinical features of the prepubertal presentations, differences in rates of AChR seropositivity, diagnostic challenges including differentiation from CMS, and response to therapy. Further studies looking at efficacy of therapies in pre- and postpubertal children are needed to better understand and support this distinct group of patients. 

## Figures and Tables

**Table 1:** Comparisons of prepubertal and postpubertal features of JMG.

	Prepubertal	Pubertal	Postpubertal/ adult
Male : female ratio	M = F	F > M 4.5 : 1	F > M 4.5 : 1

Patients with AChR antibodies detected in generalised disease	50–71% [1, 2]	68–92% [1, 2]	80–90%

Ocular presentation			
Caucasian	40% [7]	9–16% [12]	28% [6]
Chinese	75% [6]		

Progression of OMG to generalised MG	8–15% [17, 18]	23–43% [16, 19]	79% [8]

Remission (spontaneous or with treatment)	42–60% [1, 2, 20]	26% [2]	38% [9]

**Table 2:** Differential diagnosis of JMG.

Congenital myasthenic syndromes	Usually presents in infancy but can present later
Mitochondrial cytopathies	Children frequently have additional neurological impairments or epilepsy
Myopathies	Including congenital myopathies and muscular dystrophies
Neurotoxins	For example, botulism, venoms
Guillain-Barré syndrome	
Acute disseminated encephalomyelitis	
Multiple sclerosis	
Brainstem tumour	
Hypothyroidism	

**Table 3:** Treatment options in JMG.

Treatment	Evidence of efficacy in generalised JMG	Reference
Acetylcholinesterase inhibitors	First line therapy.May be sufficient in ocular JMG or mild generalised JMG	Skeie et al., 2010 [29]
Thymectomy	Recommended to increase remission rates in postpubertal, seropositive children. Not recommended in prepubertal children	Hennessey et al., 2011 [30] Rodriguez et al., 1983 [31] Tracy et al., 2009 [32] Lindner et al., 1997 [20]
Steroids	Often used in combination with steroid sparing agents. Significant side-effect profile if used long-term at high dose	Schneider-Gold et al., 2005 Cochrane review: one JMG study, others adult or unspecified age ranges [33] Zhang et al., 1998 [6]
Steroid sparing agents		
Azathioprine	Usually used in combination with corticosteroids. Occasionally used alone.	Mertens et al., 1981 [34] includes some children but no subgroup analysis Gold et al., 2008 [35] Palace et al., 1998 [36] adult study
Cyclosporin A	As monotherapy or in conjunction with corticosteroids	Tindall et al., 1987 [37] adult study Hart et al., 2009 (Cochrane) [38] series include some children
Cyclophosphamide		De Feo et al., 2002 [39] adult population Hart et al., 2009 [38] (Cochrane) series include some children
Tacrolimus		Furukawa et al., 2008 [40] Ponseti et al., 2008 [41] both include post pubertal children Ishigaki et al., 2009 [42] prepubertal female
MMF		Hehir et al., 2010 [43] includes some peri-/postpubertal children
Rituximab		Wylam et al., 2003 [44] pediatric case
IVIG/Plasma exchange		Selcen et al., 2000 [45] Gajados et al., 2008, Cochrane database [46]
